# The speed of news in Twitter (X) versus radio

**DOI:** 10.1038/s41598-024-61921-7

**Published:** 2024-05-24

**Authors:** William Brannon, Deb Roy

**Affiliations:** Center for Constructive Communication & Media Lab, Massachusetts Institute of Technology, Cambridge, MA USA

**Keywords:** Scientific data, Complex networks

## Abstract

The rapid evolution of the Internet is reshaping the media landscape, with frequent claims of an accelerated and increasingly outraged news cycle. We test these claims empirically, investigating the dynamics of news spread, decay, and sentiment on Twitter (now known as X) compared to talk radio. Analyzing 2019–2021 data including 517,000 hour of radio content and 26.6 million tweets by elite journalists, politicians, and general users, we identified 1694 news events. We find that news on Twitter circulates faster, fades faster, and is more negative and outraged compared to radio, with Twitter outrage also more short-lived. These patterns are consistent across various user types and robustness checks. Our results illustrate an important way social media may influence traditional media: framing and agenda-setting simply by speaking first. As journalism evolves with these media, news audiences may encounter faster shifts in focus, less attention to each news event, and much more negativity and outrage.

## Introduction

The speed of the news cycle and the nature of public discourse surrounding the news have important implications for civic life. If increasingly prominent new media institutions have shorter attention spans than older ones, society’s ability to sustain focus on important issues and hold the government accountable may be reduced^1^. Similarly, if outrage and negativity are intensifying as well^2^, public debate becomes more rancorous and it may be harder to reach consensus. Because different media have and serve different audiences, there may also be disparities between groups in the attention paid to those groups’ priorities.

Work in political science and computational social science has lent support to claims of an accelerating news cycle^3,4^ and of media business models becoming increasingly focused on tribalism and outrage^5^, with the latter rooted in the 1980s deregulation and the rise of talk radio^6,7^. But while both the 24-hour news cycle and outrage media have long histories, they are often claimed to have accelerated with the coming of the internet^8^, and particularly social media^2,9^. Indeed, there is even evidence that the turnover of content in social media is accelerating over time, as recent work^10^ has found for Twitter. While an increasing amount of news breaks on or is influenced by these new platforms^8^, and researchers have analyzed the dynamics of user activity within them^10^, there is relatively little hard evidence about how news cycles differ between them and traditional media. Systematic, large-scale empirical evaluations of comparative news-cycle speed and sentiment are lacking.

These questions of media dynamics matter in turn because media coverage plays an important role in determining public opinion and policy^11^, with even policymakers relying on mass media coverage for information on matters as weighty as whether to go to war (the so-called “CNN effect”)^12^. Understanding the internal dynamics of media can shed light on the influence those media have over the rest of society.

The speed of reaction to events, in particular, may be an important channel of influence for faster media on slower ones, providing a first-mover advantage that can determine the tone and framing of subsequent coverage^13,14^. These questions of inter-media influence are the subject of the extensive political science literature on “intermedia agenda-setting”^15,16^, which focuses on the effects different media and media outlets have on each other’s coverage decisions. Though our work does not directly take up such causal questions, it complements the agenda-setting literature by closely examining one potential mechanism and origin of intermedia influence.

We seek in particular to compare the nature of news propagation—its speed and affective content—across media. We compare across two major and influential communication platforms, one drawn from the domain of social media and the other from broadcast media: Twitter and U.S. talk radio (both public and commercial). (Twitter is now known as X; we refer to it as Twitter in the rest of the paper.) Both are highly impactful in their own right: radio because of its very wide reach, and Twitter because of its millions of users and a high concentration of journalists among those users.

Twitter, whose traditionally easy data availability has enabled a great deal of research^17,18^, is one of the major social media platforms with a mass audience and is widely used by journalists, playing host to many of their interactions and deliberations with each other^19,20^. Journalists themselves, while not always convinced that Twitter’s influence on their profession is salutary, have discussed it frequently and at length^21,22^.

**Figure 1:**
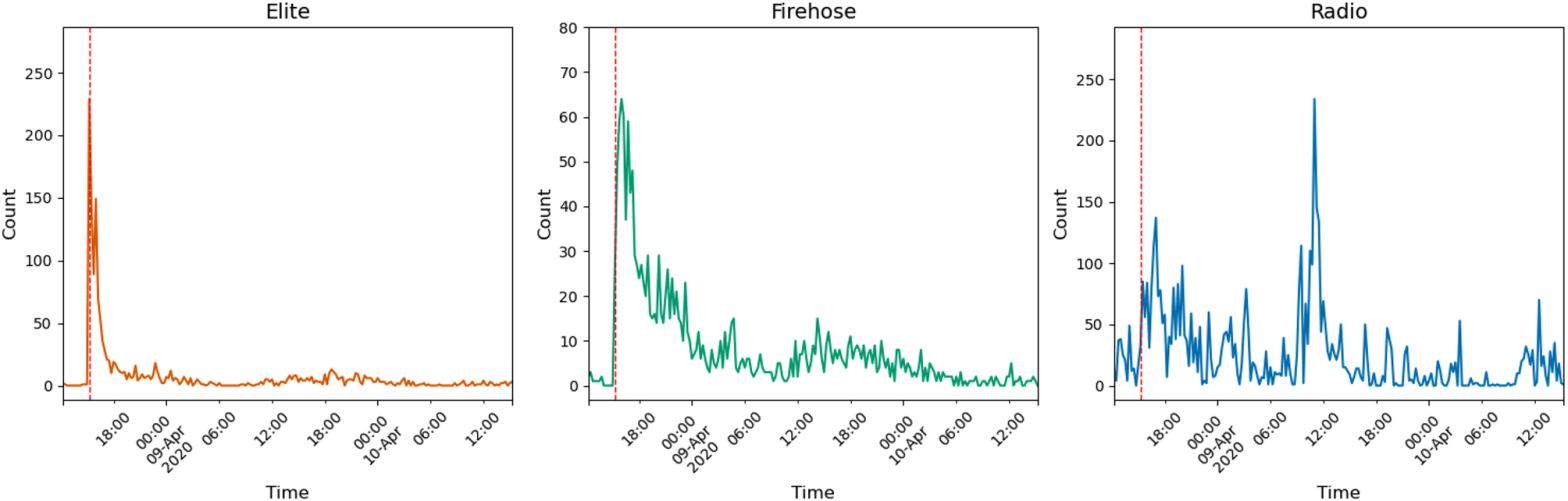
An example of one of the manually detected or keyword-based events used as a robustness check, Bernie Sanders’ withdrawal from the 2020 presidential race. Note that discussion on both elite and mass Twitter clearly peaks and falls off before radio discussion.

Radio, though less of a venue for intra-journalistic discourse, reaches as many as 88% of Americans each week^23^, and there is a long literature documenting its political influence, especially over conservatives. Yet it receives less attention than its substantial influence on political discourse and individual behavior^6,24,25^ suggest it deserves. Much of this lack of attention is due to the difficulty of obtaining corpora of audio or transcripts, which we address by building the comprehensive radio transcript corpus discussed below.

It is not clear, however, exactly how we should expect news events’ rise, decay and sentiment to differ between radio and Twitter. On the early, rising side of a news event, a finding of more rapidly rising Twitter discussion would be in line with qualitative and theoretical literature on the “24-hour news cycle,” the acceleration of news^3,8^, and Twitter’s role in it^26,27^, with our study testing and contributing empirical evidence to this literature.

There has been much less quantitative work, however, on the falling side—the decay of attention to news. Some studies have examined the time course of traffic to online news stories^28,29^, or engagement with them on sites like Digg^30^, showing that attention to such stories decays rapidly. Others, such as Hu et al.^31^, and Lin et al.^26^ have focused on Twitter and the dynamics of attention on the platform during events. But these studies have focused more on the rise of attention to an event than its fall, and have used small numbers of hand-selected events. Pfeffer et al.^32^ analyzed the “half-life” of a tweet and found that engagement with the typical tweet falls off rapidly, but this work examines individual tweets rather than events and defines engagement via impressions rather than, as we do, discussion. Closer to our work is Liu et al.^33^, which examines the “screen-persistence” of events detected in Twitter by an in-house event detection system. This work, however, also defines persistence by presence in the Twitter feed rather than discussion, looks only at very short-term (<1 h) persistence, and does not make cross-medium comparisons. We go beyond prior work by making a systematic and cross-medium study of the question of attention decay in entire news events.

We operationalize attention as discussion, rather than audience exposure to content^32,33^, for reasons of data availability. We had access to radio broadcasts and tweet-level information, but not to radio audience data or the impression data needed to measure exposure on Twitter. This common situation^34^ focuses our analysis on the behavior of the media elites who produce news and those engaged users who explicitly comment on it, and means that the results have greater implications for research on agenda-setting than on media effects. While the hypothesis that audience attention rises and decays in line with discussion is plausible, and our results with Twitter’s firehose below provide some evidence for it in that medium, we cannot say for sure. On the agenda-setting side, though, because past media discussion can frame and influence future coverage^13,14^, a medium whose engagement with an issue both begins and ends sooner may have a greater ability to set the agenda.

It is not, however, obvious which medium we should expect to see move on more quickly. Twitter’s highly interactive nature, which allows co-creation of the news^35^ and a stronger feedback loop between journalists and audience, may cause events to proceed faster and conclude more quickly than on a one-to-many broadcast medium like radio. On the other hand, there are also reasons to expect events on the radio to be less persistent. Unlike genuinely ephemeral radio broadcasts, which are not generally recorded, easily available after the fact, or searchable, tweets persist online and can be shared by users, perhaps prolonging event lifespans. Interactivity and virality may also be a two-way street, with a story that strikes a chord with users persisting longer than journalists alone would have continued discussing it. Finally, because the two media can interact, we might find that they have similar attention spans. With discussion on Twitter keeping an issue alive and sparking interest on radio (or the reverse), news events may wind down in a similar way across media.

As for sentiment, questions of affect have also been extensively studied on Twitter and social media in general, with a particular focus on outrage. Prior work has examined its psychology^36^, mechanisms behind outrage ‘firestorms’^37^, the distribution of outrage^38^, and many case studies^39,40^, which generally concur with everyday experience that outrage is prominent and influential on Twitter. Outrage in traditional media has also received substantial attention, especially in conservative media^41^, with pioneering studies from Sobieraj and Berry^5,42^ finding very high levels of outrage on cable news and talk radio.

We have not, however, been able to identify a large-scale comparison between outrage or other affective states on Twitter and in traditional media. The closest prior work^5,42^ compared talk radio and cable news to political blogs, rather than social media, and found both very high levels of outrage on radio and cable, and *lower* levels in blogs.

There is some support, in other words, for believing that either Twitter or radio (the medium of hosts like Rush Limbaugh and Sean Hannity) might be more outraged and negative; comprehensive data are lacking. One contribution of our work is to fill in this lacuna with an apples-to-apples comparison between the two media, especially one which allows us to examine the distribution of outrage and negativity over the lifecycle of an event.

We address these questions with three large datasets, one of radio broadcasts and two providing different views of Twitter. Our analysis shows that Twitter, as a medium, differs from radio in having a systematically shorter attention span for news than radio, and in reacting to it in a more outraged way. These biases, likely rooted in properties of the media themselves, suggest an important connection between platform affordances and news cycle dynamics. Identifying specific mechanisms relating platform features to news cycle behavior may be an important direction for future research.

## Results

### Datasets

Our three datasets each cover a total of 6 months, spread over 3 years: September–October 2019, March–April 2020, and January–February 2021, encompassing important news events including President Trump’s first impeachment and the start of the COVID-19 pandemic. We refer to these three datasets—radio, elite Twitter, and firehose Twitter—interchangeably as “datasets” or “corpora,” to distinguish them from the “media” of Twitter and radio.

**Radio.** The first is a novel and very large dataset of transcribed radio broadcasts, developed using a similar approach to Beeferman et al.^43^. Our overall radio corpus comprises 517,895 h of audio from 228 talk and public radio stations, including content from 1066 shows and amounting to about 5.2 billion words of text. We perform extensive quality filtering at both the station and show levels to exclude irrelevant content, such as sports and music, and poor transcriptions. To avoid double-counting widely syndicated shows, we also deduplicate this corpus at the show level, picking one station per day to represent each episode of a syndicated show. After these steps, we have 89,203 hours of content from 144 stations and 810 shows, amounting to about 902 million words of text.

**Table 1:** Counts of automatically detected events by year and corpus.

Year	Firehose	Elite	Radio
2019	224	393	41
2020	203	566	53
2021	73	105	36

**Elite Twitter.** Our second dataset consists of 2.6 million tweets from a diverse set of 2834 elite journalists and politicians (“elite Twitter”) posted during the study periods. Parts of our analysis also rely on the follow-graph edges between these users. The set of users includes leading figures in journalism and politics, including every member of Congress and reporters from the major national news outlets. It also includes 203 radio hosts and staff for whom we could identify Twitter handles. Both radio and elite Twitter datasets thus offer different views of the social and professional milieu surrounding radio hosts, making analysis involving these two datasets an even more direct comparison of the two media themselves. The Supplementary Information tests this assumption in detail, showing that the two media encode similar social and information-spreading structures^17^.

**Firehose Twitter.** The third and final dataset encompasses 24.0 million tweets sampled randomly from Twitter’s firehose of all tweets (“firehose”). Because the firehose dataset is drawn from the general Twitter user base, using it in addition to elite Twitter allows us to ensure that we are measuring effects of the medium as such, rather than only the behavior or occupational conventions of journalists. If we find similar behavior for both, as in general we do, this behavior is likely to stem from Twitter’s affordances and properties as a medium.

### Event detection

The first step in comparing event lifecycles is concretely defining an ‘event.’ As in much existing literature on news event detection (e.g.,^33^), we define events here in a media-first, but also medium-agnostic, way: An event is a subject of discussion and attention, rising to prominence in the discourse before falling off. Concretely, our events are thus groups of related *items* (tweets or radio speaker turns), which allows us to use the same definition of and detection method for events in both media, without relying on medium-specific features like retweets. This definition captures a wide range of news, both clearly exogenous events and those whose timing is driven by journalists’ editorial choices.

**Figure 2:**
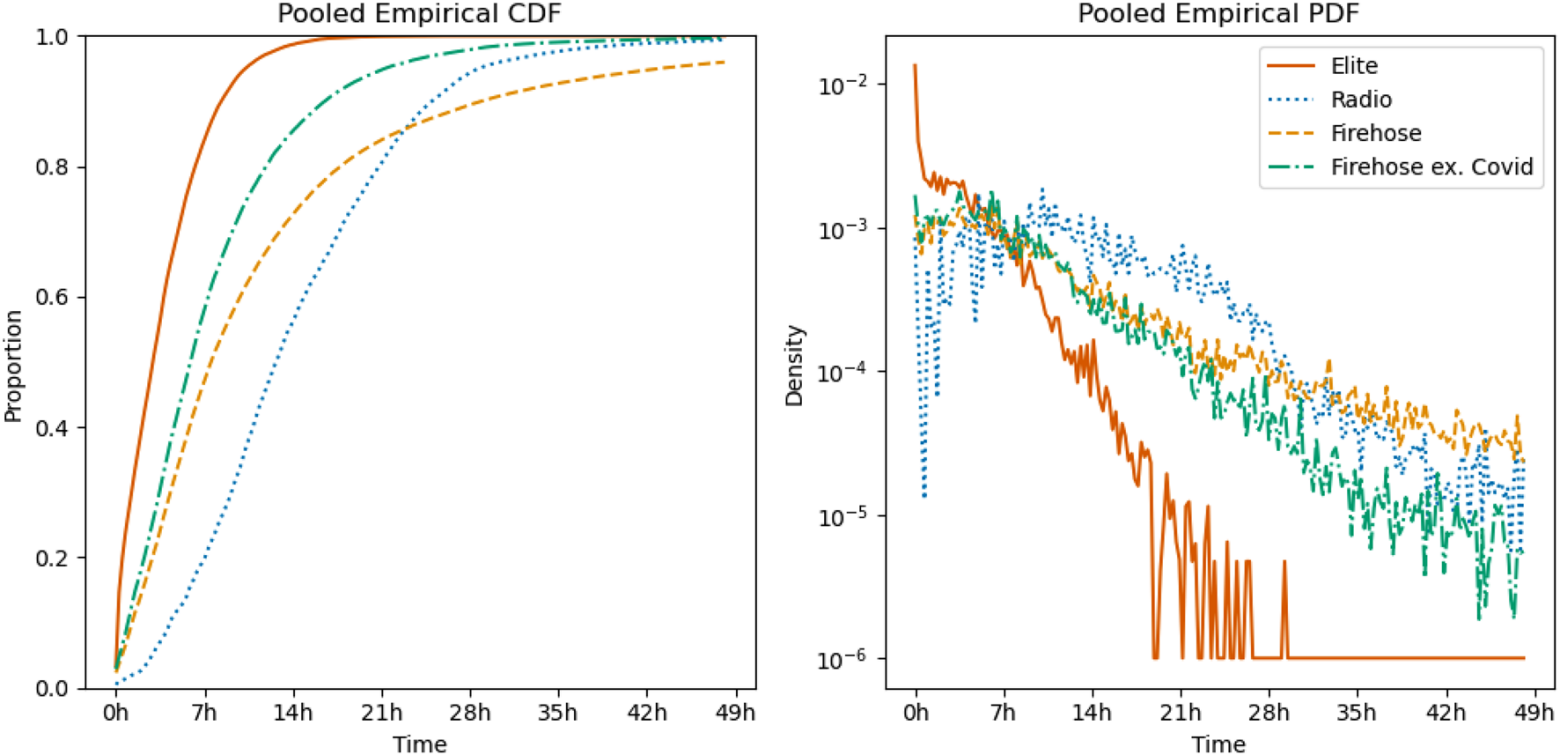
The average empirical CDF and PDF of mentions of the automatically detected events. We find the time after event start of each item (tweet or radio speaker turn) contained in the event, compute the empirical CDF at 15-min resolution and average the CDFs over the events. The PDF is obtained by a central difference estimate of the derivative of the CDF. Only mentions occurring within 48 h of the event are included. The density of Twitter discussion shows the same rapid rise and rapid fall, first exceeding and then falling below radio, as in the manual case. The “Firehose” line represents all firehose stories, while the “Firehose ex. Covid” line excludes Covid-related stories from March and April 2020.

To detect these events, we use the newsLens system^44,45^, following Zhang et al.^46^ in using Sentence-BERT^47^ for item embeddings. Our newsLens detection process identified 1694 total events, broken down by year and corpus in Table 1. In light of recent findings that patterns of user behavior on Twitter are changing over time^10^, we break down much of the following analysis by year as a robustness check.

### Event lifecycles

We first look at the average empirical CDF and PDF of *within-event relative times*. Considering an event to start at the time of its first item (tweet or speaker turn), an item’s relative time is its offset from its event’s start time. If, for example, a tweet assigned to some event occurs exactly 100 s after the event starts (i.e., with its first tweet), the tweet’s relative time is 100 s.

The average eCDFs and ePDFs are shown in Fig. 2, and demonstrate the general point of faster Twitter lifecycles clearly: discussion on elite Twitter rises fastest, followed by the firehose, with both remaining at a higher relative rate than radio for a time, before both falling below the level of radio discussion for a longer time as events wind down.

Here, however, there is a complication: As shown in Fig. 2, firehose discussion of Covid in March and April of 2020 does not follow this pattern. We identified Covid-related events by embedding a probe phrase (“pandemic of coronavirus disease 2019”), measuring the cosine similarity of the phrase’s embedding to the centroid of each event’s item embeddings, and selecting those with similarity above an empirical threshold of 0.1. Such events in the firehose rose more quickly than radio, but did not fall off as quickly as events in other years or (in 2020) about other topics. Inspecting these events revealed the reason: Firehose discussion of the pandemic at this point was constant, closely related to people’s lived experience of the virus and countermeasures, rather than particular news stories, and difficult to separate into discrete events. This unusual behavior during an exceptional time for the media ecosystem throws into relief the more typical behavior, during other years and on other topics in 2020, of firehose discussion winding down faster than radio.

Examining the events concretely, we find that the cumulative fraction of elite Twitter discussion (i.e., the empirical CDF value) is greater than the cumulative fraction of radio discussion at every 15-min query point out to 48 h after event start. The eCDF of firehose discussion, if we exclude the Covid-related events from 2020, is also strictly between elite Twitter and radio at all query points. Two-sided percentile-bootstrap tests find that all of these differences are significant at the $5\sigma$ level ($p < 2.86 \cdot 10^{-7}$), except for the last 14 hours of firehose/radio differences. (All but the last three hours are significant at the lower $p = 0.05$ level.) Though not shown in Fig. 2, results are similar for 2019, 2020 and 2021 subsets.

For another view of event onset and decay, we calculate means and standard deviations (SDs) of the relative times, in seconds, for each event. A later average relative time indicates an event whose center of mass, so to speak, occurs further after event onset (i.e., not rising as quickly), while a higher SD indicates one which is more spread out in time (i.e., not decaying as quickly). The relative-time means and SDs tell a similar story to the eCDF/ePDF analysis. The average within-event time is significantly lower at the $5\sigma$ level for both elite Twitter events ($t = -\,35.07$, df = 1192, $p < 2.86 \cdot 10^{-7}$) and firehose events excluding 2020 Covid-related events ($t = -\,10.24$, df = 463, $p < 2.86 \cdot 10^{-7}$) than radio events. SDs are also significantly lower for elite events ($t = -\,28.06$, df = 1192, $p < 2.86 \cdot 10^{-7}$) and firehose events excluding 2020 Covid-related events ($t = -\,5.26$, df = 463, $p < 2.86 \cdot 10^{-7}$) than radio events. All tests are two-sided independent-samples *t*-tests. Results are similar if broken down by year.

**Figure 3:**
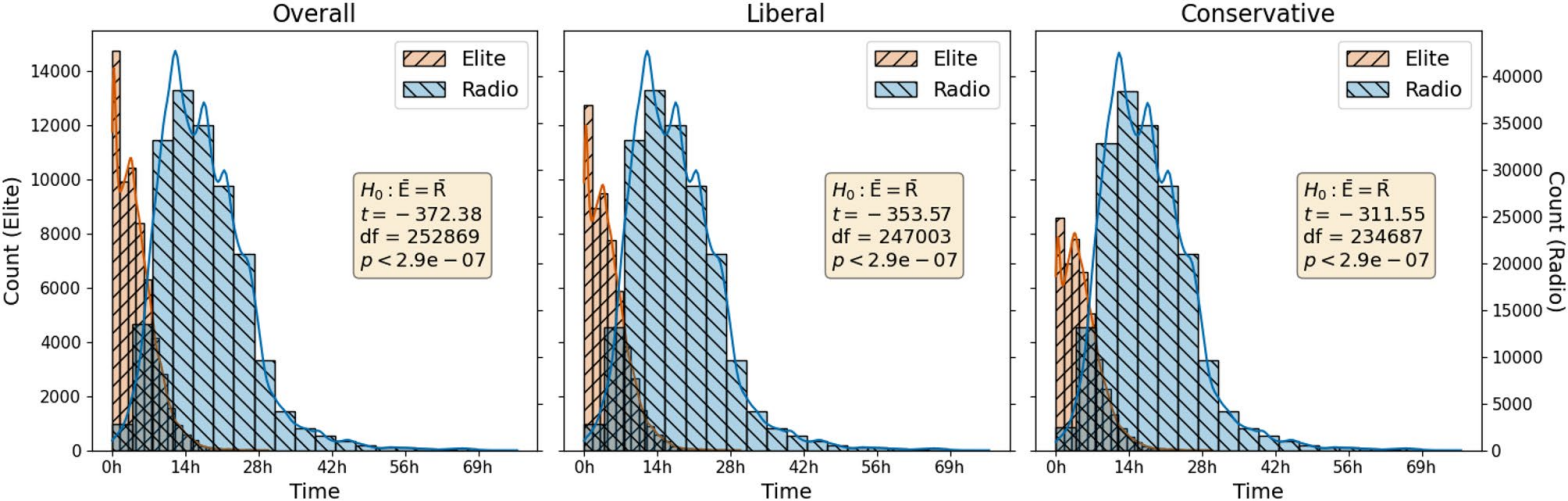
The distribution of within-event relative times of items: tweets for events on elite Twitter, and speaker turns for events on the radio. The average piece of discussion occurs earlier on Twitter than radio, and the average event winds down faster. Both effects are significant at the $5\sigma$ level, the difference in means by two-sided independent-samples *t*-tests as shown in the text boxes, and the difference in standard deviations (not shown) by two-sided bootstrap tests.

For elite Twitter and radio, we show the distributions of within-event relative times at the item level in the leftmost pane of Fig. 3. (See below for the other two panes). There are clear and large differences in both mean and SD between Twitter and radio, both of which are statistically significant at the $5\sigma$ level ($p < 2.86 \cdot 10^{-7}$). We use a two-sided independent-samples *t*-test for the difference in means and a two-sided bootstrap test for the difference in SDs.

In addition to the breakdowns by year above, we conduct several additional robustness checks: matching events across media for a paired comparison, breaking results down by ideological affiliation, and examining an additional set of manually detected events identified by keywords. An example manually detected event, Bernie Sanders’s withdrawal from the 2020 presidential race, is shown in Fig. 1. These checks, on different subsets and even with different event-detection methods, reinforce the conclusions of the main analysis: Discussion of the typical event on Twitter both rises to its peak and falls off much faster than on the radio.

### Event matching

First, we match the detected events within year across our three datasets, to detect events corresponding to the same real-world occurrence, and also examine event onset and decay on this set of (elite, radio, firehose) triples. Details of the matching process are given in the “Methods” section.

Matching yields 38 triples totaling 114 events. Results on these events are quite similar to those on the broader corpus. This time using two-sided paired *t*-tests, average within-event times are once again significantly lower for elite Twitter ($t = -\,6.80$, df = 37, $p < 2.86 \cdot 10^{-7}$) and firehose events ($t = -\,3.54$, df = 29, $p = 0.001$) than for radio events, as are SDs for elite ($t = -\,7.37$, df = 37, $p < 2.86 \cdot 10^{-7}$) and firehose ($t = -\,2.33$, df = 29, $p = 0.027$) Twitter. Comparisons to the firehose exclude triples whose firehose member is a 2020 Covid-related event. The empirical CDFs (omitted for space) similarly show elite Twitter discussion rising and falling faster than radio, with firehose discussion in between.

### Manually detected events

As an additional check, we identified a set of events from Wikipedia, detailed in the “Methods” section, which occurred during the same periods as the 2019 and 2020 portions of our datasets, and tracked mentions of them by counting keywords.

**Figure 4:**
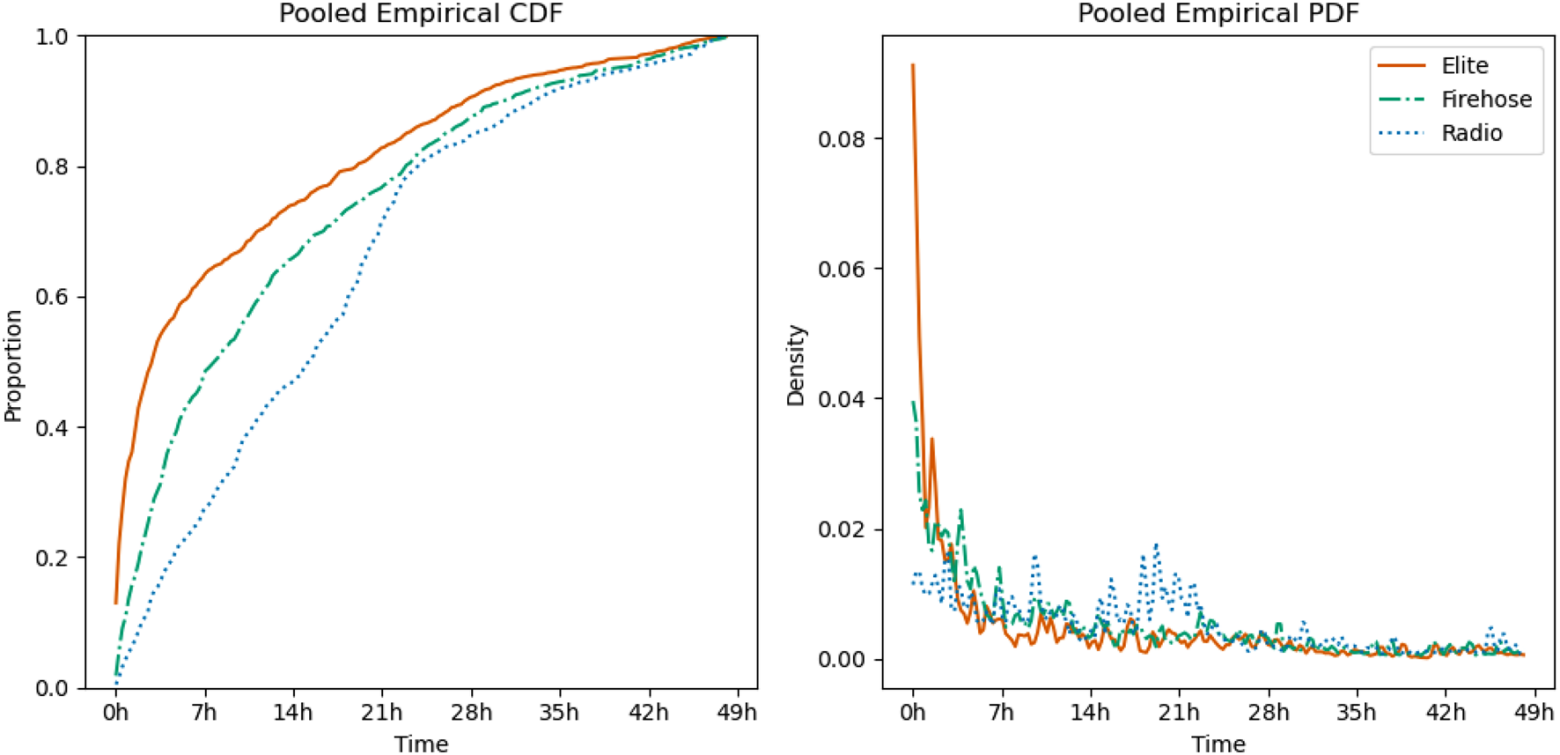
The average empirical CDF and PDF of keyword mentions of the 10 manually detected events. For each event in each corpus, we find the time after event occurrence of each mention, compute the empirical CDF at 15-min resolution and average the CDFs over the 10 events. The PDF is obtained by a central difference estimate of the derivative of the CDF. Only mentions occurring within 48 hours of the event are shown, and we count each mention rather than each piece of content. Note the clear pattern of a spike of early elite-Twitter discussion, with the firehose lagging a bit behind, rapidly decaying into a period of more discussion on the radio than in either segment of Twitter.

Results overall are quite similar to those found with the newsLens-detected events. Figure 4 summarizes the time courses of the events, and shows that as in Fig. 2, elite Twitter discussion both rises and falls fastest, followed by the firehose, with radio responding slowest. We also examined relative-time means and SDs. To avoid drift in the real-world events associated with our keywords, only mentions within 4 days of event start are considered. In 9 of 10 cases, except for Purdue Pharma’s bankruptcy, Twitter discussion has a lower average relative time than radio discussion. Over all 10 events, the average within-event time of a mention for elite Twitter is 65,133 s, vs 100,065 for radio, a difference of 53.6%. The SDs show a similar pattern, with 8 of 10 being larger for radio than for Twitter. The average of the event-level SDs of Twitter events’ relative times is 83,412 s, while on radio the analogous figure is 92,063 s, a 10.4% difference.

Finally, we looked by hand for newsLens events which matched manually detected events. Because news stories can be large and complicated, with many degrees of freedom in dividing them up, not all manually detected events could be matched to newsLens events. We were able to identify three events, however, in both Twitter and radio. John Bolton’s firing as National Security Advisor, the announcement of the 2019 Trump impeachment inquiry, and Elizabeth Warren’s withdrawal from the 2020 presidential race all showed similar patterns through keyword counts to what we found by automatic detection: discussion on Twitter rising sooner and falling faster than on the radio.

### Ideological differences

We also examined whether event lifecycles differ between the liberal and conservative sides of Twitter and radio. Ideology is one of the most important organizing principles of both radio^6^ and political Twitter^48^, and differences in event dynamics would point to differences between the ideological sides in how the two media are organized.

We used approaches described in the “Methods” section to identify liberal and conservative content on both Twitter and radio. Lacking a good way to make ideological inferences about firehose users, we consider only elite Twitter and radio. We used these ideological assignments to break each event into two sub-events for the liberal and conservative sides of its overall discussion, excluding events whose liberal or conservative sides were below a certain minimal size threshold. We were left with 912 liberal events and 624 conservative events, each out of 1694, with a total of 965 unique events represented between them.

The results are summarized (at the item level) in the rightmost two panes of Fig. 3. For both liberals and conservatives, Twitter discussion tends to be shorter and finish faster than on radio. Both effects for both ideological groups are statistically significant at the $5\sigma$ level ($p < 2.86 \cdot 10^{-7}$), but are slightly stronger for liberals than conservatives, perhaps reflecting a greater role for Twitter in liberal discourse. The empirical CDFs (not shown for space) tell a similar story to the elite and radio curves in Fig. 2, with Twitter discussion rising and falling faster than on the radio for both liberals and conservatives, and for all three years.

Results are also similar if the manually detected events are broken down by ideology. Of the 20 liberal or conservative versions of the 10 events, 18 have a higher average within-event time for radio than for Twitter, and most also have a higher SD of within-event times on the radio side than on Twitter.

### Affective biases

Turning from the lifecycles of events to their affective content, we next aim to test whether affect differs between Twitter (elite and firehose) and radio. To detect affect, we follow Yin et al.^49^ in using a neural language model^50^ fine-tuned for natural language inference to assign affect scores to each tweet or radio speaker turn in the newsLens events. We examine three affect metrics—negativity, emotionality and outrage. Our analysis includes the 2020 Covid-related firehose events; results are similar if they are excluded.

**Figure 5:**
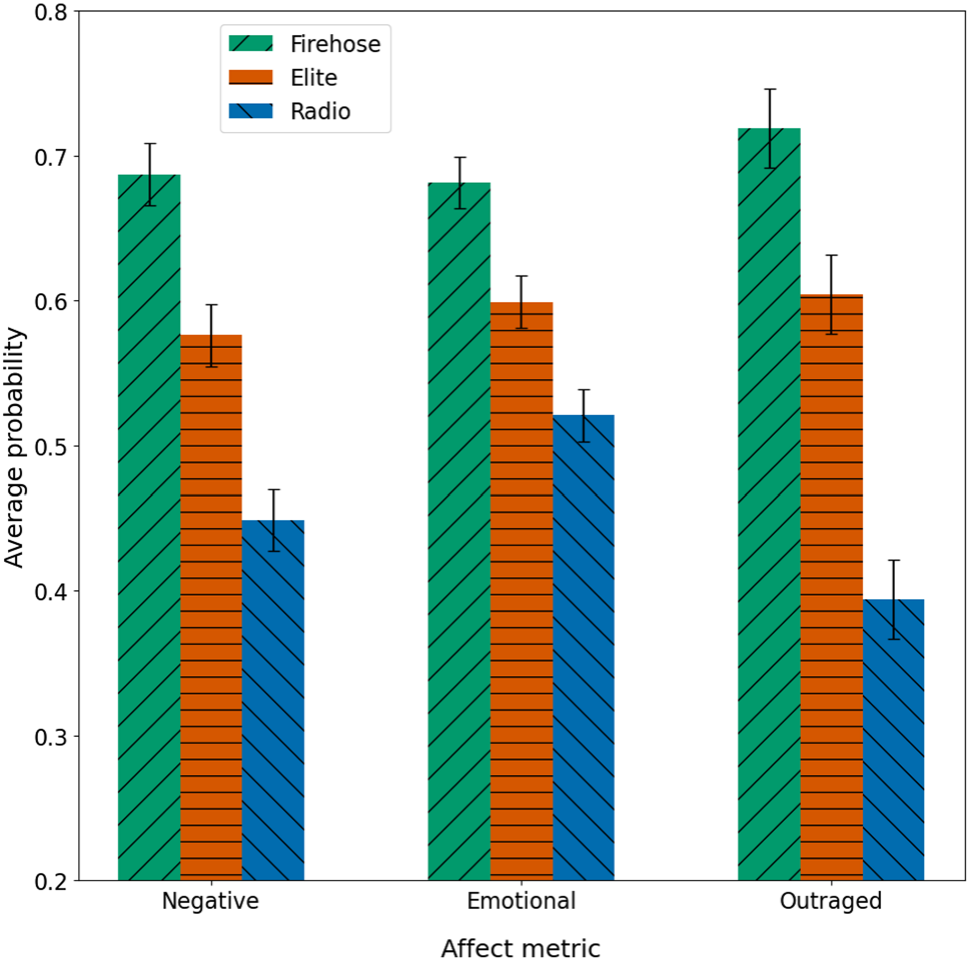
Story-level average scores for three affect metrics. The underlying item-level scores are probabilities, and range from 0 to 1. 95% confidence intervals are shown. All inter-corpus differences are significant at the $5\sigma$ level ($p < 2.86 \times 10^{-7}$) by two-sided Mann-Whitney U-tests.

The results are shown in Fig. 5, averaging item-level scores up to the event level. (Results at the item level are similar.) We find large differences on all three affect measures, always in the same direction: Firehose Twitter is most negative, emotional and outraged, while radio is least, and elite Twitter is in between. All inter-corpus differences are statistically significant at the $5\sigma$ level ($p < 2.86 \cdot 10^{-7}$) by two-sided Mann-Whitney U-tests.

### Outrage dynamics

We also find strong, consistent and statistically significant differences between media in the evolution of affect over an event’s lifecycle. This analysis again includes the 2020 Covid-related firehose events, and the results are again similar if they are excluded. We test for linear time trends, and especially inter-corpus differences in these trends, for all three affect metrics, but focus our discussion especially on negativity and outrage, which are of greater theoretical interest^5^. “Emotional,” which can also involve positive emotions, is not closely correlated with the other two scores: Item-level emotionality has a 0.364 correlation with outrage and 0.260 with negativity, while the correlation between outrage and negativity is 0.729.

**Figure 6:**
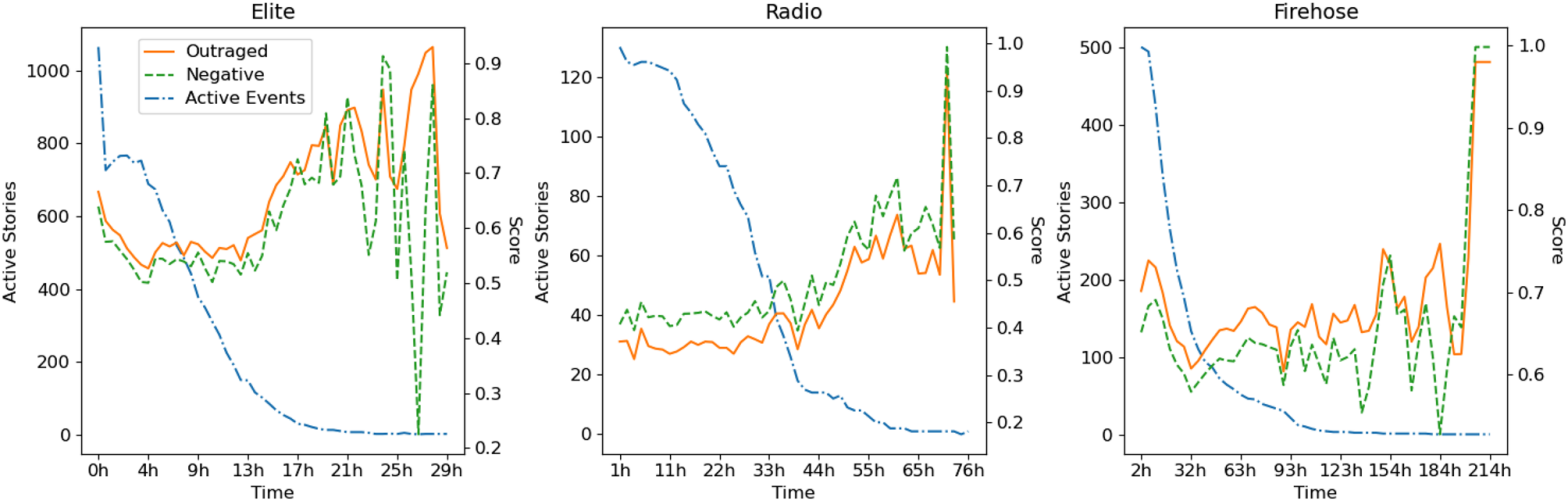
Average levels of negativity and outrage over the event lifecycle. We also show the number of detected events which are active (i.e., contain tweets or radio speaker turns) at each point in the lifecycle. These figures are calculated by dividing relative time into 200 equal-sized bins (i.e., half-percentiles), within which we average negativity and outrage scores and count the number of active stories. There are notable differences between all three corpora, but especially between radio and the two Twitter datasets. The firehose events shown here include 2020 Covid-related events; without them, the distribution of outrage and negativity is similar but the number of active stories falls off much more quickly.

Figure 6 shows average negativity and outrage levels over time, revealing substantial differences between our corpora, and between radio and Twitter. Notably, there is one clear commonality across the three datasets: A few very long-lasting events are especially outraged. Out of 1694 events, 51 persist past 24 h on elite Twitter or 48 h in the other two corpora, and content after this point is 17% more negative and 27% more outraged than earlier content.

For deeper analysis, we fit logistic regression models to the data for each corpus, predicting the affect scores as a function of linear trends in within-event relative time. (The item-level scores are probabilities, so a linear model of their log-odds is a logistic regression.) Between three affect metrics and three media, we fit 9 models and apply corresponding Bonferroni corrections to hypothesis tests. Results are shown in Table 2a.

All six models for negativity and outrage have statistically significant linear time trends at the Bonferroni-corrected $5\sigma$ level ($p < 3.18 \cdot 10^{-8}$), using both here and below the usual two-sided *t*-tests of the parameters. The signs, however, differ between radio and Twitter. Both negative and outraged affect show declining trends (negative coefficients) for elite and firehose Twitter, and an increasing trend (positive coefficients) for radio. Emotionality is similar in finding significant declining trends for elite and firehose Twitter, but no significant trend—i.e., a stable level over time—on the radio.

To test our main question of whether these effects are significantly different from each other, rather than only from 0, we fit additional models. We took pairs of corpora (i.e., firehose/elite, elite/radio, radio/firehose) and modeled item-level affect scores as a function of relative time, corpus and the interaction. All of the interaction terms are significant at the Bonferroni-corrected $5\sigma$ level, indicating significant differences in event lifecycles between media.

But these patterns leave open an important question: Are the differences because of time trends within individual events, or because of differences between media in how long different kinds of events last? We tested this question with further regression analysis, fitting the same models as above but with event fixed effects. Results are shown in Table 2b. If most variability is at the event level, with time trends coming from differences correlated with outrage and negativity in how long events persist, we should expect now not to find significant time trends or differences in time trends. And indeed we do not; neither any within-event time trends nor any inter-corpus differences are significant at the Bonferroni-corrected $5\sigma$ level after including event fixed effects, and usually have much smaller magnitudes. The observed patterns in the distribution of affect, including the lack of a pattern for emotionality on the radio, are almost all due to differences (or the lack thereof) at the event level.

## Discussion

We examined how news event lifecycles differ between two prominent communications media: Twitter and talk radio. Leveraging large-scale, comprehensive data from both media, we find that Twitter has a systematically shorter attention span than radio, with discussion of events not only beginning sooner but also decaying faster. These findings are robust, with similar results in all three years, among liberals and conservatives, within pairs of events matched across media, and using an additional set of manually identified events. Twitter is also much more negative, emotional and outraged than radio, with differences both in the average levels of these affect variables and in their dynamics over time. Outrage and negativity in particular are more fleeting on Twitter, with the differences in dynamics driven by differences at the event level in how long more and less outraged events persist.

The scale and representativeness of the datasets leading to these conclusions are an important contribution of our analysis: Far from covering only one station, show, or Twitter account, we can take a systematic look at a major broadcast medium and compare it to both elite media actors and a mass audience on social media. Indeed, including both sides of Twitter allows for a particularly stark demonstration of medium-specific effects: We find that even among the general Twitter user base (i.e., the public rather than the press), news discussion rises and falls faster than among professionals on the radio.

**Table 2:** Results from regression models predicting affect (indicated in the first column of the tables with “Emo” = emotional, “Neg” = negative, and “Out” = outraged).

(a) Linear main effects of relative time, with and without event fixed effects				
Affect	F.E.	Firehose	Elite	Radio
Emo.	N	**− 0.153** (0.003, $p < \hspace{0.35em} p_{5\sigma }$)	**− 0.160** (0.006, $p < \hspace{0.35em} p_{5\sigma }$)	0.009 (0.004, $p = 0.04$)
	Y	− 0.023 (0.026, $p = 0.37$)	0.010 (0.015, $p = 0.51$)	0.000 (0.015, $p = 0.99$)
Neg.	N	**− 0.071** (0.007, $p < \hspace{0.35em} p_{5\sigma }$)	**− 0.113** (0.014, $p < \hspace{0.35em} p_{5\sigma }$)	**0.074** (0.007, $p < \hspace{0.35em} p_{5\sigma }$)
	Y	0.076 (0.054, $p = 0.16$)	− 0.034 (0.025, $p = 0.16$)	− 0.018 (0.030, $p = 0.55$)
Out.	N	**− 0.074** (0.006, $p < \hspace{0.35em} p_{5\sigma }$)	**− 0.106** (0.012, $p < \hspace{0.35em} p_{5\sigma }$)	**0.104** (0.006, $p < \hspace{0.35em} p_{5\sigma }$)
	Y	0.051 (0.044, $p = 0.24$)	− 0.073 (0.022, $p = 0.00$)	0.006 (0.025, $p = 0.79$)

(b) Interaction effects of relative time and corpus from models fit on pooled pairs of corpora with relative time main effects, a corpus dummy and the interaction effect. Dummies are for elite in the first column and radio in the other two (i.e., the estimates are for the difference in time trend going from firehose to elite, from elite to radio, and from firehose to radio)				
Affect	F.E.	Elite vs. Firehose	Elite vs. Radio	Radio vs. Firehose
Emo.	N	**− 1.098** (0.055, $p < \hspace{0.35em} p_{5\sigma }$)	**0.425** (0.020, $p < \hspace{0.35em} p_{5\sigma }$)	**0.162** (0.012, $p < \hspace{0.35em} p_{5\sigma }$)
	Y	0.104 (0.123, $p = 0.40$)	− 0.026 (0.044, $p = 0.55$)	0.021 (0.049, $p = 0.67$)
Neg.	N	**− 0.815** (0.112, $p < \hspace{0.35em} p_{5\sigma }$)	**0.376** (0.035, $p < \hspace{0.35em} p_{5\sigma }$)	**0.280** (0.023, $p < \hspace{0.35em} p_{5\sigma }$)
	Y	− 0.350 (0.202, $p = 0.08$)	0.069 (0.067, $p = 0.30$)	− 0.121 (0.101, $p = 0.23$)
Out.	N	**− 0.761** (0.097, $p < \hspace{0.35em} p_{5\sigma }$)	**0.393** (0.029, $p < \hspace{0.35em} p_{5\sigma }$)	**0.369** (0.020, $p < \hspace{0.35em} p_{5\sigma }$)
	Y	− 0.628 (0.180, $p = 0.00$)	0.197 (0.065, $p = 0.00$)	− 0.027 (0.082, $p = 0.74$)

Coefficients are given first, with standard errors and *p*-values after in parentheses. Models with event fixed effects are marked with ’F.E.’ = Y and use standard errors clustered at the event level. Effects significant at the Bonferroni-corrected $5\sigma$ level ($p < 3.18 \cdot 10^{-8}$), under the usual two-sided *t*-tests of the parameters, are in bold and show $p < p_{5\sigma }$. Relative time variables were centered and scaled prior to fitting.

These differences are likely to be rooted in each medium’s affordances for discussion^51^, and especially in how social media’s affordances differ from those of a broadcast environment^52^. The clearest example we find of a tie to specific medium affordances is in the event-level origin of time trends in affect. Our results show that radio and Twitter differ in how hospitable they are to different kinds of stories, leading emotionality, negativity and outrage to be less persistent (but more intense) on Twitter and more persistent (but less intense) on the radio.

The average levels of affect variables also suggest connections to platform design and affordances. We find that the Twitter firehose is the most outraged, followed in turn by elite Twitter and then by radio. In light of recent findings^53^ that elite incivility on Twitter may spread by a reinforcement process, driven by positive feedback from the audience, our results support the idea that audience demand may be an important factor in the higher level of outrage in elite Twitter than on the radio. The underlying affordance is then that on Twitter, journalists and media personalities can get audience feedback easily and very rapidly, much more so than for radio. We cannot, however, say for certain that these audience dynamics are responsible, because of the lack of audience engagement data. It is, rather, a plausible hypothesis to be investigated in future work.

Another valuable subject for future work is which (if any) useful intervening variables exist between platform affordances and high-level discourse phenomena like sentiment. Differences in news cycle speed, for example, may lead to more opinionated content in one medium than another (because opinion is easier to produce rapidly than reporting or interviews), leading in turn to more outrage. We have not explored such effects, including the difference between news and opinion, in part because of the difficulty of operationalizing and measuring opinion in media without a clear editorial separation between it and news. Doing so would, however, be a good subject for future work.

Despite connections to affordances, our findings also rebut a simplistic view of content as entirely determined by properties of media. Firehose discussion of Covid in 2020 deviated sharply from the usual patterns, reflecting the power of real-world events to make themselves felt. The medium may be the message^51^, but not the only message. Determining more precisely how affordances influence both the speed and sentiment of the news cycle is an important subject for future research, including in media other than those we consider here.

The recent sale of Twitter also makes this work timely. There are an increasing number of similar platforms, based on real-time sharing of text posts, which gives us good reason to think the conclusions may apply more broadly. And yet at the same time, the reduced availability of Twitter data for research has made it harder to observe journalists’ interactions with each other on social media. Using data from the last period when this activity was concentrated and readily observable, we can still provide a comprehensive investigation.

Overall, our results contribute substantial empirical evidence to the literature on news cycles and their determinants, including the first systematic comparison between outrage on Twitter and traditional media. They also illustrate an important channel of influence Twitter and its propensity for outrage may have on other media: framing and agenda-setting by the simple expedient of being the first to speak.

Media’s ongoing shift toward digital formats provides a final reason these results matter. If journalism and media continue to shift to online venues, the social media biases we find here may become more influential. The fast forgetting and proclivity to outrage we find characterize Twitter may thus be not only features of the present, but a preview of the future.

## Methods

### Data sources

**Radio data.** On the radio side, we relied on a large dataset of stations, shows and their content, collected using a procedure similar to that of Beeferman et al.^43^. In brief, we began with data from the third-party company Radio-Locator (https://radio-locator.com/) on the complete list of U.S. radio stations, selected about 200 of those with talk or public-radio formats and online streams of their broadcasts, and continuously downloaded the broadcast streams. The exact number of stations varied over time with technical issues and additions or removals for other projects.

We performed ASR in two steps: First, given the large scale of the corpus, we converted all of the streamed audio to text using an in-house Kaldi-based^54^ speech recognition system to reduce costs. These transcripts are used in the keyword-based manual event detection. Second, we retranscribed the random sample used in automated detection (discussed below) with OpenAI’s Whisper model^55^ to obtain better performance.

We also collected information on the stations’ schedules (i.e., start and end times of their programs) by scraping their websites, using this to label transcribed broadcasts with the show of origin. Because station websites are not always updated promptly, however, these labels are sometimes out of date. In total, the corpus included transcribed radio broadcasts, divided into approximate speaker turns, the web-scraped show labels, and station metadata. The overall dataset, or ‘raw’ corpus, comprises 517,896 hours of audio from 228 talk and public radio stations, with 1,066 shows represented, amounting to about 5.2 billion words of text.

To exclude irrelevant content and ensure quality, we performed several filtering and deduplication steps:

- We excluded any audio for which we could not collect schedule data. Note that a large share of this content should be syndicated programming, and will be represented by collection of the same content on other stations.
- We manually reviewed each of the 1066 shows and excluded those which did not contain any news discussion, such as music call-in shows, gardening shows, and most sports talk, as well as the BBC World Service, which rarely discusses US politics and is a poor match to elite Twitter. A handful of entire stations which had changed to music formats during our collection periods were also excluded.
- We dropped some airings (an “airing” is a show/station/date combination) to ensure quality, specifically those with ASR or schedule-data confidence scores below empirically chosen thresholds.
- Finally, we deduplicated the remaining airings across stations to “episodes,” or show/date combinations, to avoid over-weighting large syndicated shows. We did so by choosing the airing for each episode with the highest average schedule and ASR confidence.

This process yielded a cleaned or ‘final’ corpus of 89,203 hours of content from 144 stations and 810 shows, amounting to about 902 million words of text.

**Twitter user selection.** We collected data from the Twitter API on 2834 accounts. These accounts were chosen to represent a set of elite journalists and politicians, relying in part on lists from news organizations, and in particular included hosts and/or production staff of certain radio shows. We refer to the whole set as “elite Twitter.”

We first collected Twitter accounts for hosts or production staff of certain shows included in the radio data. Of the shows recorded in the final radio corpus, we selected 68 for which to collect host/staff Twitter accounts. The shows we selected included the largest 45 syndicated shows (i.e., shows recorded on multiple stations) and then approximately half again as many local shows. We then manually collected matching Twitter accounts: If accounts of hosts (i.e., on-air talent) were available, we included them; for producers and staff, we preferred staff in senior positions or with a public presence, including more junior staff in a few cases where no other Twitter accounts could be found. In total we collected 203 Twitter accounts for these 68 shows, with 65 shows having accounts that posted tweets during the study periods.

While the final set of 203 radio-linked accounts is not based on a random sample of all shows, it is reasonably representative of the largest and most important shows (because it includes most of them) without neglecting smaller programs. For example, the syndicated shows include the most popular and influential shows from both public and conservative radio during this period: for example, All Things Considered and Morning Edition on one side, Rush Limbaugh and Sean Hannity on the other.

In selecting the other journalists and politicians included in the sample, we aimed to cover a range of geographies and ideological perspectives. Among politicians, we accordingly included Twitter accounts for every member of Congress (via C-SPAN’s members-of-congress Twitter list) and a small number of particularly prominent political figures who were not in Congress at the time of data collection (e.g., Donald Trump, Barack Obama, and Joe Biden). Among journalists, we mainly deferred to the decisions on notability of news organizations themselves, collecting most of our users from eight Twitter lists of prominent reporters. Two of these lists, from C-SPAN (“cspan/political-reporters” and “cspan/congressional-media”), included journalists regardless of ideological affiliation, while two further lists each covered left-leaning (“slate/left-leaning-tweets” and “msnbc/msnbc-hosts”) and right-leaning (“slate/right-leaning-tweets” and “foxnews/shows-hosts”) journalists. We also included lists of prominent journalists from the New York Times (“nytimes/nyt-journalists”) and the Washington Post (“washingtonpost/washington-post-people”). As with politicians, we supplemented these lists with a small number of manually selected commentators or institutional accounts who were not on any of these lists. This process produced 2631 Twitter accounts for journalists and politicians.

**Elite Twitter data.** For each Twitter user, we collected all tweets posted by that user during the study periods, the list of IDs of other users that user follows and is followed by (the “follow graph”), and certain account metadata. (The 203 radio users’ tweets were collected only during the 2019 and 2020 periods.) For analysis other than ideology detection (discussed below), we used information only about our list of 2834 users, and discarded follow edges to users not on the list. Not considering second-degree connections through other users focuses our graph on users’ explicit choices, and the preferences or interests they reveal. The ideology analysis and Supplementary Information do, however, use follow edges to these other users to calculate an ideology measure. We do not collect tweets from followers or followees not in the list of 2834 elite-Twitter users. In total we collected about 2.6 million tweets.

**Table 3:** The 10 manually detected events included in our analysis.

Event	Date	Time
John Bolton Fired	2019-09-10	16:00:00
Purdue Bankruptcy	2019-09-16	03:15:00
Tom Brady Free Agent	2020-03-17	12:45:00
Shane Gillis Fired	2019-09-16	20:00:00
Manning Released	2020-03-12	21:15:00
Huffman Sentencing	2019-09-13	18:00:00
Bernie Drops Out	2020-04-08	15:15:00
NBA Season Cancelled	2020-03-12	01:30:00
Warren Drops Out	2020-03-05	15:30:00
Trump Impeachment	2019-09-24	18:30:00

All times are UTC and are for the 15-min period start time immediately preceding the event.

We pulled the follow graph once, in early November 2019, and pulled tweets every day during the three two-month study periods. Using these tweets, we also generated the mention graph, in which there is a directed edge from A to B if A has mentioned B’s username in a tweet. (We use the mention graph for analysis in the Supplementary Information, not in the main paper.) After creating both follow and mention graphs, we collapsed all users in each graph associated with a given show to one node for that show.

**Firehose data.** To collect a random sample of all tweets during our study period, we rely on Twitter’s Decahose API^56^, which provides a 10% random sample of all tweets. We collected two separate samples, one for analysis with manually detected events (8.4 million tweets) and another for analysis with automatically detected events (15.6 million tweets), totaling 24.0 million tweets.

### Automatic event detection

Our analysis of automatically detected events employed the newsLens system^44,45^ for news event detection to find events in the elite Twitter, firehose Twitter and radio corpora. We ran the detection process separately on each combination of year and corpus, such as 2021 firehose data. Separating the media during the detection phase produced notably higher-quality detected events, and we address its downside of no longer having paired events with the matching process described below.

The system has several stages, or modules: first, we compute the similarity of each item to every other item within a sliding window over the corpus. (Recall that an “item” is a tweet or radio speaker turn.) We use a window of length 16,000 s. We follow Zhang et al.^46^ in using the cosine similarity of Sentence-BERT embeddings^47^ rather than bag-of-words representations or doc2vec to compute the inter-item similarity in the first stage of newsLens, building on their finding of better downstream performance. Only item pairs with similarity above a configurable threshold are kept; we used a value of 5 standard deviations above the mean for all years and corpora, with (year, corpus)-specific values for SD and mean.

The selected item pairs form a graph, and we next use the Louvain community-detection algorithm^57^ to decompose it into events, or news stories. Because our graphs were not in general fully connected, we ran the Louvain algorithm separately within each component of at least 300 items. To detect events which are interrupted and then resume, detected events which do not overlap in time are merged together if the cosine similarity of their centroids exceeds an empirical threshold; we used the 99.9th percentile of all pairwise similarities.

Because the radio and firehose corpora are much larger than elite Twitter, for simplicity and a fair comparison we first randomly downsampled the final radio corpus to the same size as elite Twitter before running newsLens. Because the firehose contains a large amount of content which is not about news, such as Twitter memes and discussion of movies or music not released recently, we downsampled the firehose corpus to six times the size of the elite Twitter corpus (rather than the same size) to get enough news-related events.

To exclude radio and firehose content which was not about news (e.g., advertising, weather reports, Twitter memes), we filtered out detected radio and firehose events whose maximum cosine similarity to any elite event was below a manually tuned threshold of 0.6—the same threshold across all years and corpora. We also discarded any detected event consisting of fewer than 10 tweets or speaker turns, in order to exclude a set of apparently spurious detections with very few items.

### Manual event detection

We identified a set of events from Wikipedia^58,59^ which occurred during the same periods as the 2019 and 2020 portions of our corpora. We aimed to collect a particular kind of event, namely one which (a) got more than a trivial amount of discussion on both radio and Twitter, (b) was readily identifiable by simple keywords, and (c) occurred at a specific point in time rather than extending over a longer period. We collected the first 5 events each during the 2019 and 2020 corpus periods that we found to meet these criteria, for a total of 10. Using point events of this sort allows us to avoid considering the time course of the event itself, as with, say, a hurricane. Our events can thus all be viewed as announcements: of firings, bankruptcies, court decisions, etc. To avoid drift in the real-world events associated with our keywords, only mentions within 4 days of event start are considered. The events we identified are shown in Table 3.

### Event filtering

Given these automatically detected events, we had the further problem of ensuring they were about news. Both radio and the Twitter firehose contain some discussion of other subjects: on the radio, things like weather reports and advertising; in the firehose, such content as memes and discussion of movies or music not released recently. Elite Twitter, composed as it is of journalists and politicians, almost exclusively discusses news. Some of these non-news discussions can have lifecycles resembling those of news events and result in spurious detections by our newsLens algorithm.

We thus discarded non-news events in radio and the firehose according to a filtering strategy, the overall idea of which is to drop any radio or firehose event whose maximum cosine similarity to any elite event is below a certain threshold. We used the same threshold across all years and corpora. The first step was to compute the centroid of each event’s tweet or speaker-turn embeddings. We then calculated the cosine similarity of all pairs of events which both overlapped in time and were in different media (i.e., elite/radio, elite/firehose, or firehose/radio). We kept all elite events, because of elite Twitter’s near-exclusive focus on news, and kept also any radio or firehose event whose cosine similarity to any elite event was above a threshold value. After manual inspection, we found that a value of 0.6 was effective at excluding non-news content without dropping too many news events.

We filtered in this way, relying on elite Twitter, for two reasons. First, because we are interested in comparing properties of Twitter and radio as media, it is more important to have a common set of news events than to have an entirely comprehensive one. Minor news events which were discussed only on mass Twitter or local radio would be important in a thorough survey of the news agenda on these media, but we are interested in the different question of comparative news cycle behavior. Avoiding confounders like topical variation can help. Second, deferring to a large set of national journalists on what the news is greatly reduces researcher degrees of freedom in this regard.

### Event matching

Starting from the filtered set described above, we selected matching (elite, radio, firehose) triples as follows. As before, we first generated the centroid of each event’s tweet or speaker-turn embeddings, and then calculated the cosine similarity of all pairs of events which both overlapped in time and were in different media (i.e., elite/radio, elite/firehose, or firehose/radio).

We then found (elite, radio) matches. The first step was to discard pairs which failed to satisfy some quality constraints: (a) if either event in the pair had fewer than 30 items, (b) if the events differed by a factor of more than 3 in their wall-clock duration, or (c) if the events differed by more than a factor of 7 in the number of included items. Using the resulting set of event pairs, we then formed an undirected graph over these events, with one edge per pair, and used the cosine similarity as an edge weight. Finally, we took the maximum-weight matching^60^ as implemented in the networkx package for Python^61^ to find the most similar pairs of events.

We next used a similar procedure to match these pairs to firehose events, applying the same quality filters and forming a graph of firehose events connected to those elite and radio events which had already been matched. Edges were again weighted by cosine similarity. The same maximum-weight matching procedure then yielded (elite, firehose) and (radio, firehose) pairs, with each firehose event matched to the closest elite or radio event. We then grouped each firehose event with the (elite, radio) pair to whose member it had been matched, forming (elite, radio, firehose) triples.

Finally, we filtered the resulting triples by dropping those below a threshold in either (elite, firehose) or (radio, firehose) similarity, with the same threshold for both similarities. After manual inspection, we found that a threshold of 0.5 produced a high-quality set of events.

### Ideology detection

We identify liberal and conservative content in both the elite Twitter and radio corpora, relying on particular properties of each medium to do so. Lacking a good way to make ideological inferences about users in the general firehose, we ignore it here and consider only elite Twitter and radio.

**Twitter.** To identify liberal and conservative sides of Twitter, we rely on Louvain communities^57^ in the follow graph, which has been shown to incorporate both social and discursive aspects relevant to a phenomenon like ideology^17^. Community detection returns a small set of communities covering the entire graph, which upon inspection have clear interpretations. One consists mainly of conservative journalists and politicians, while others contain mostly liberals; we considered tweets from the first community’s members to be conservative and all others to be liberal. The Supplementary Information examines these communities in more detail and substantiates our interpretation.

**Radio—methodology.** For radio, it is less clear how to identify an ideological dimension. Lacking any labeled training data, we cannot follow Vijayaraghavan, Vosoughi, and Roy^62^ in using a classifier to infer user ideology. In lieu of Bayesian methods, such as^63^, we instead adopt a simpler approach (also used, for example, in^64^ and^65^) based on applying dimensionality reduction to Twitter’s follow graph.

We first form the bipartite graph between radio accounts, on the one hand, and the set of all Twitter users (not just our set of journalists and politicians) who follow at least two radio accounts. (Here, unlike in the rest of the analysis, we rely on the full set of follow graph edges pulled for the elite-Twitter accounts rather than only the edges among the elite-Twitter users themselves.) From the adjacency matrix of this graph, which has radio accounts as columns and followers as rows, we compute the pairwise cosine similarity matrix of the radio accounts. We can then project the radio accounts into a lower-dimensional space (we use 2D for easy visualization and inspection) via classical multidimensional scaling (MDS). In a heavily ideological set of users like ours, where distances largely reflect ideological homophily, one of these dimensions is interpretable as latent ideology.

Because of properties of MDS, the scores are only well-defined up to a change of sign. We found higher scores to be more conservative and lower scores to be more liberal, which our discussion reflects. (The scores range from about − 0.75 on the liberal end to +1.25 on the conservative end.) To get scores at the radio-show level, we averaged the ideology scores of the radio accounts associated with each show to obtain the show’s ideology score. Next, we map these show-level scores back to radio content. Any content from a show with an ideology score receives that show’s score, and other content on stations with such shows (i.e., content from other shows for which we do not have Twitter handles) receives the average score of shows with ideology scores. In all, this process assigns scores to 81.0% of the radio data. The remaining radio content within each event is excluded from our ideology analysis. Finally, to get a dichotomous indicator for ideology, we consider any radio content whose ideology score is higher (i.e., more conservative) than the mean to be conservative and other content to be liberal.

**Radio—validation.** The follow graph-based measure of ideology validates well, and is readily interpretable. On the radio side, we evaluate it at the show level, taking as reference labels whether a show airs more on public radio or non-public, commercial radio, because of the well-known fact that non-public, commercial talk radio skews heavily conservative politically^6^. (According to our schedule data, all but one show airs at least 99% on one or the other.) We find that the continuous ideology scores achieve an AUC of 0.933 at predicting these public/non-public labels. More qualitatively, Twitter users with the highest or most conservative scores include well-known right-wing hosts like Mark Levin, Sean Hannity, and Dana Loesch, while the lowest or most liberal scores belong to users who are affiliated with NPR. Other reassuring patterns the scores reflect include variation by geography (radio stations in urban areas tend to be associated with more liberal shows) and frequency band (AM talk is a particularly conservative genre). They also line up well with the follow-graph communities: The community we found to contain most conservative hosts has the highest average score by far at 0.605, compared to − 0.09 for the centrist community and − 0.529 and − 0.607 for the two liberal communities.

As a robustness check, we also evaluated the simple option of considering statements from public radio stations to be liberal and statements from other stations to be conservative (relying on the well-known average political leanings of commercial talk and public radio^6^), and found very similar results.

**Event splitting.** We use these ideological assignments to break each event into liberal and conservative sub-events for its discussion in the appropriate parts of radio and Twitter. We drop from the liberal side any event without at least 10 liberal items, and analogously on the conservative side, and also drop from both sides any event without at least 20 items in total.

### Affect metrics

We measure item-level negativity, emotionality and outrage with an approach^49^ based on natural-language inference (NLI). The NLI task takes a “premise” and a “hypothesis,” and estimates probabilities that the premise entails, contradicts, or is independent of the hypothesis. In more detail:

- We begin with a BART-Large model^50^ fine-tuned for NLI (https://huggingface.co/facebook/bart-large-mnli).
- For each label (“negative,” “emotional,” or “outraged”), we take the text of the input item (tweet or speaker turn) as premise, and the sentence “This example is [label].” as hypothesis.
- The model’s NLI head produces probabilities of entailment, contradiction and independence. We take the entailment probability as the probability that the input item satisfies the label, discarding the other two estimates.

A hypothetical example might have the premise “@username this is a terrible tweet, delete this” and the hypothesis “This example is negative,” with the model estimating P(entailment) of 0.96. We would then take this value as the tweet’s “negative” score. Manual spot-checks reveal that this procedure produces reasonable scores for both tweets and radio content.

### Affect dynamics

This section describes the various logistic regression models we fit to analyze the time dynamics of affect. We state the models here for one arbitrary affect variable *A*, because the specifications are identical except for the choice of *A*.

We have a set of *N* items (tweets or speaker turns) which have been assigned to detected events, indexed by a variable *i*, each of which has a parent corpus $K_i \in \{\text {E}, \text {F}, \text {R}\}$ (for elite, firehose and radio). Write $C \triangleq \{\text {E}, \text {F}, \text {R}\}$ for the set of corpora, $I^{(c)}$ for the set of items in corpus *c*, $\text {ind}(I^{(c)})$ for the indices of items in $I^{(c)}$, and finally $S^{(c)}$ for the set of events detected in corpus *c*.

Each item in each corpus *c* also has a within-event relative time $t_i \ge 0$, an event ID $S_i \in S^{(c)}$ indicating which event it was assigned to, and an affect variable $A_i \in [0, 1]$, interpretable as a probability of the item displaying the underlying affect state.

For notational simplicity, let $L_i \triangleq \log (A_i / (1 - A_i))$ be the log-odds of affect, $D_i(s) \triangleq \mathbbm {1}\{S_i = s\}$ be a dummy variable for whether item *i* is in event *s*, and $G_i(c) \triangleq \mathbbm {1}\{K_i = c\}$ another dummy for whether item *i* is in corpus *c*. The $\epsilon _i$ terms below are error terms.

**Single-corpus models.** First, we model the log-odds of affect as a linear function of relative time:

1 $$\begin{aligned} L_i = \beta _1 t_i + \beta _0 + \varepsilon _i \end{aligned}$$

for all $c \in C$ and $i \in \text {ind}(I^{(c)})$.

Next, we test for time trends net of event-level effects by modeling affect as a linear function of relative time, controlling for event fixed effects (note that we cluster the standard errors by event):

2 $$\begin{aligned} L_i = \beta _1 t_i + \sum _{s \in S^{(c)}} \beta _s D_i(s) + \beta _0 + \varepsilon _i \end{aligned}$$

again for all $c \in C$ and $i \in \text {ind}(I^{(c)})$.

**Multi-corpus models.** To test for inter-corpus differences, we take pairs of corpora (elite/firehose, elite/radio and firehose/radio) and model them as linear functions of relative time, corpus, and the interaction effect. For corpora $c_1, c_2 \in C$, we have:

3 $$\begin{aligned} L_i =&\beta _3 t_i + \beta _2 G_i(c_1) + \beta _1 t_i G_i(c_1) + \beta _0 + \varepsilon _i \end{aligned}$$

where $i \in \text {ind}(I^{(c_1)}) \cup \text {ind}(I^{(c_2)})$. The coefficients $\beta _1$ then indicate the magnitude of the inter-corpus difference in relative-time trend.

As above in the single corpus case, we also fit models with event fixed effects, again clustering the standard errors by event. For linear relative time, we have:

4 $$\begin{aligned} L_i =&\beta _3 t_i + \beta _2 G_i(c_1) + \beta _1 t_i G_i(c_1) + \sum _{s \in S^{(c)}} \beta _s D_i(s) + \beta _0 + \varepsilon _i \end{aligned}$$

where $$i \in \text {ind}(I^{(c_1)}) \cup \text {ind}(I^{(c_2)})$$.

### Supplementary Information

Supplementary Information 1.

## Data Availability

Pursuant to our agreement with Twitter, Inc. (recently renamed X Corp.), we release a list of tweet IDs for the set of tweets used in the study. The complete radio data supporting our findings are available from the authors for research purposes. For copyright reasons, we are unable to make the entire radio dataset public. To assist with replication, however, we release certain derived data for radio, including the detected events, statistics about them, and item-level information other than the textual content of the radio broadcasts. Tweet IDs and radio data are available at https://doi.org/10.6084/m9.figshare.24454777. Further questions about data availability or requests for access should be directed to William Brannon <wbrannon@mit.edu>.

## Code availability

Our code to prepare these datasets and carry out the analysis is available at https://doi.org/10.6084/m9.figshare.24546400.
