# Supplementary material for: The speed of news in Twitter (X) versus radio
Source: Sci Rep. 2024 May 24;14:11939. doi: 10.1038/s41598-024-61921-7 (PMC11126604; doi:10.1038/s41598-024-61921-7)
Supplement: Supplementary file 1 — Supplementary Information 1. [file 41598_2024_61921_MOESM1_ESM.pdf]

Supplementary Information for "The speed of news in Twitter (X) versus radio"

Correspondence: wbrannon@mit.edu

We focus here on demonstrating that elite Twitter and radio represent similar underlying social and information-disseminating structures. We would expect them to: Both media are part of the same tightly coupled world of political and journalistic elites, and ought to reflect a common (partially offline) social world. Several analyses find exactly this, providing reassurance that slower decay on radio is an effect of the medium rather than an artifact of differences between our datasets. Our results are not, therefore, just about measuring differences between who is active on these media.

In order to get a closer match between the collection times of radio speech and the Twitter follow graph, the analysis below uses only 2019 and 2020 data.

Coairing-Follow Similarity

We first attempt to compare the elite Twitter follow and mention graphs to the *coairing graph* for radio, in which two shows are connected if they air on the same radio station (within a week of each other, a restriction intended to minimize the impact of errors in webscraped schedule data). This graph, which does not depend on the content of shows, reflects corporate syndication and programming decisions, and we view it as an observable analog of the Twitter graphs. It embodies the same social and informational aspects [1] as Twitter: Hosts whose shows air on the same station are more likely to have professional connections than random host pairs, and corporate programmers have clearly judged that such shows are similar enough to be of interest to the same audience. If we were comparing to follow or mention graphs involving the shows' audiences, the relationship might plausibly run the other way, with shows similar on Twitter because they air on the same stations and thus acquire the same listeners. Using Twitter data for elites instead mitigates this confounder.

The full coairing graph contains 785 nodes, one for each show in the 2019/2020 portion of the final radio corpus, connected by 21,927 edges. The graph has two connected components: one with 11 shows, from a radio station in Detroit which airs only local content, and another with the other 774 shows from all other stations. To allow a clean comparison with Twitter, we analyze only the Twitter-matched coairing graph: the subgraph over shows which have been matched to Twitter handles, all of which are in the larger component. For brevity, we refer to this graph simply as "the coairing graph" below. There are 67 Twitter-matched shows in total.

The coairing graph has quite similar structure to both the follow and especially the mention graphs. In addition to the summary statistics presented in Table 1, we demonstrate this similarity with the SimRank node similarity algorithm [2]. SimRank generates similarity scores for pairs of nodes in a graph, with nodes more similar the more they occur in similar contexts in the graph. Figure 1 compares SimRank scores for all pairs of nodes in the coairing graph with two other sets of SimRank scores from the follow and mention graphs over radio shows. (Recall that we aggregated the follow and mention graphs over the 203 radio-linked Twitter accounts, or "radio accounts" for short, up to the level of shows.) Coairing SimRank is strongly correlated with follow and especially mention SimRank, much more so than in a configuration-model baseline, highlighting that shows are in similar social and informational positions on Twitter and on air.

Statistic	Coairing	Follow	Mention
Order (# nodes)	56	55	52
Size (# edges)	434	370	343
Average degree	15.50	13.45	13.19
Transitivity	0.67	0.53	0.57
Avg. Clust. Coef.	0.77	0.60	0.65

Table 1. Selected summary statistics of the follow, mention, and coairing graphs for the set of Twitter-matched shows, demonstrating a substantial degree of similarity. These statistics cover the large connected component of each graph, omitting a few shows out of the full 67 which were isolates.

In particular, coairing SimRank is strongly bimodal, with two clear clusters visible above and below a threshold value of about 0.15. Manual inspection suggests these clusters divide along left-right ideological lines: similar pairs are mostly those where both shows are conservative or both are liberal. Follow-graph and especially mention-graph SimRank, while less ideologically polarized, replicate much of this structure.

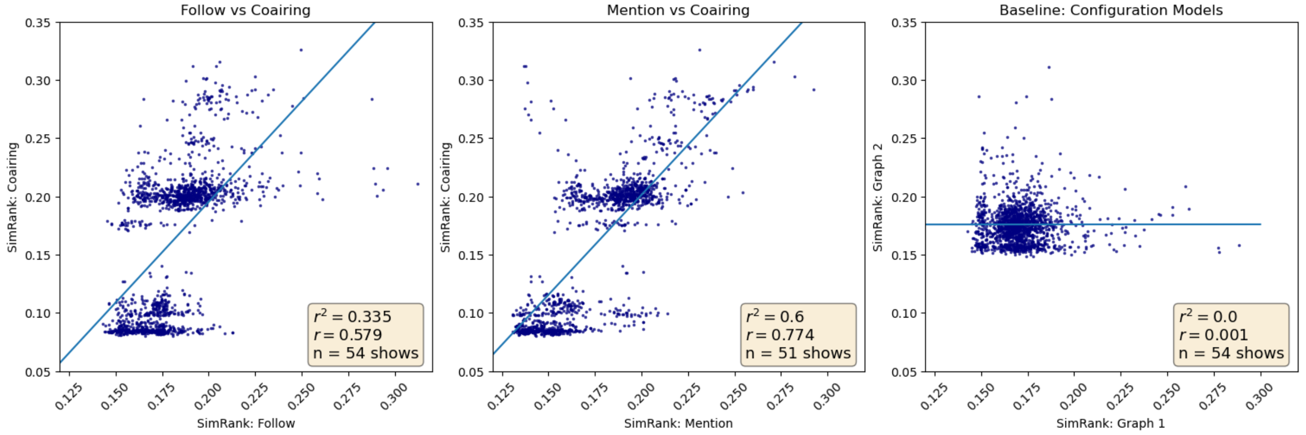

Figure 1. Relationships between SimRank similarity scores for all pairs of non-isolated nodes in the follow, mention and coairing graphs on shows. The leftmost two plots compare coairing SimRank to follow-graph and mention-graph SimRank; the rightmost plot illustrates a baseline comparison of two configuration-model graphs having the same degree distributions as the follow and coairing graphs. Note that because SimRank is uninformative for nodes with no edges, all comparisons exclude show pairs in which either show was an isolate in either graph being compared. (Isolates are, concretely, shows whose associated Twitter accounts followed or were followed by no other shows' accounts, and analogously for mention and coairing graphs.) The follow-coairing comparison thus includes 54 shows or nodes out of 67, while the mention-coairing comparison includes 51; note that a larger sample of shows would be expected to include more social context and produce fewer isolates. For comparability with the undirected coairing graph, we ignored edge directions in computing follow and mention SimRank scores. One outlier show pair has also been excluded from each of the two left panels (though not the r^2 calculations) for ease of visualization.

Predicting Structure From Show Content

We can further highlight the close connections between the two media by comparing Twitter to the content of radio – the same content that makes the medium influential in the first place. We extract certain social attributes from the Twitter follow graph (Louvain communities and a latent ideological dimension), transfer them to radio via the mapping between shows and the Twitter accounts of their hosts and staff, and predict them from the content of the broadcasts. The predictive methodology is deliberately simple; rather than using a neural language model, we rely on n-gram models. Doing so underscores the point that Twitter and radio share a common underlying structure; the signal from one medium about the other is not subtle or difficult to find.

Community structure

First, as described in the Methods section, we examine the community structure of the elite-Twitter follow graph. Discarding edge directions, we use the Louvain algorithm [3], which does not require specifying the number of communities to find. The algorithm identifies four communities with modularity 0.326, of roughly equal size, which are readily interpretable as centrists, conservatives and two groups of liberals from New York and DC. The importance of New York and DC here reflects both the geographic concentration of national journalism in these cities and the users included in the sample, with large numbers of accounts from the New York Times, the Washington Post and the US Congress.

These communities are well represented on the radio. However, community detection in the follow graph between the radio accounts only, without elite Twitter at large, performs poorly and yields uninterpretable communities with very low modularity. We consider this phenomenon further evidence of a link between the two media: Twitter includes the social context necessary to make sense of radio.

Latent ideology

Political ideology is a very important organizing principle of both the modern radio ecosystem [4] and political Twitter. In the Twitter case, the reason is homophily in the evolution of the follow graph: users are likely to follow those who are similar to themselves, especially politically similar [5]. We adopt the same approach to ideology detection, based on multidimensional scaling, as used in the main text.

Prediction

We first excluded all radio episodes (recall that an "episode" is a show/date combination) present in the final corpus which came from shows without at least one matching Twitter account. This left us with 3,850 episodes. We split the radio data into a

75% training set and a 25% test set at the airing (show/date) level, clustering the randomization by show so that every airing of a given show ends up in the same split of the data.

Target	Metric	Score
Communities	AUC	0.942
Ideology	R^2	0.621
Binarized Ideology	AUC	0.879

Table 2. Out-of-sample performance of models predicting show-level ideology and follow-graph community from episode text. The AUC value for "communities" is the simple average AUC.

For feature selection, we considered all unigram and bigram features, ordered them by term frequency, and selected the top 20,000, also applying a tf-idf transformation. Two types of features were excluded, however. First, we dropped about 250 terms which would have allowed simply memorizing the link between shows and Twitter data, including the names of shows, hosts and stations — for example, it's not interesting or useful to learn that the term "Hannity" is a marker of a conservative show. Second, we excluded features which were present only in data from 2020. Given that the 2020 data covered March and April of that year, this restriction was intended to focus the prediction task on persistent features of radio, rather than on the details of early COVID-19 coverage. To avoid overfitting, only the training set was used in the selection of features.

To predict ideology and community from these features, we used generalized linear models: logistic or softmax regressions for discrete outcomes, and a linear model for continuous ideology. The predictors were first centered and scaled to have zero mean and unit variance. We relied on scikit-learn [6] for most of these steps.

The classification and regression results are summarized in Table 2. Performance in community prediction is substantially better than chance, reaching a simple average AUC of 0.942.
Ideology models performed comparably well, with both a linear model of ideology as a function of text and a dichotomized version, predicting whether a show's ideology score exceeded the overall mean, demonstrating strong out-of-sample predictive performance. Moreover, an examination of the most predictive n-grams demonstrates that the models learn sensible relationships. Some of the unigrams and bigrams, for example, most characteristic of high (conservative) ideology are "threatening", "extinction" and "our american", while "the environment" is predictive of low (liberal) ideology.

References

[1] H. Kwak, C. Lee, H. Park, and S. Moon, "What is Twitter, a social network or a news media?" In *Proceedings of WWW 2010*, New York, New York, USA: ACM Press, 2010, p. 591. DOI: 10/c2k8cj.

[2] G. Jeh and J. Widom, "SimRank: A measure of structural-context similarity," in *Proceedings of KDD 2002*, Edmonton, Alberta, Canada: ACM Press, 2002, p. 538. DOI: 10/c4h8xv.

[3] V. D. Blondel, J.-L. Guillaume, R. Lambiotte, and E. Lefebvre, "Fast unfolding of communities in large networks," *Journal of Statistical Mechanics: Theory and Experiment*, vol. 2008, no. 10, P10008, 2008. DOI: 10/bxz74q.

[4] B. Rosenwald, *Talk Radio's America: How an industry took over a political party that took over the United States*. Cambridge, Massachusetts: Harvard University Press, 2019.

[5] Y. Halberstam and B. Knight, "Homophily, group size, and the diffusion of political information in social networks: Evidence from Twitter," *Journal of Public Economics*, vol. 143, pp. 73–88, 2016. DOI: 10/f88stf.

[6] F. Pedregosa, G. Varoquaux, A. Gramfort, *et al.*, "Scikit-learn: Machine learning in Python," *Journal of Machine Learning Research*, vol. 12, no. 85, pp. 2825–2830, 2011. [Online]. Available: http://jmlr.org/papers/v12/pedregosa11a.html.
